# CRISPR/dCas9‐mediated activation of multiple endogenous target genes directly converts human foreskin fibroblasts into Leydig‐like cells

**DOI:** 10.1111/jcmm.14470

**Published:** 2019-07-02

**Authors:** Hua Huang, Xiangyu Zou, Liang Zhong, Yanping Hou, Jin Zhou, Zhiyuan Zhang, Xiaoyu Xing, Jie Sun

**Affiliations:** ^1^ Department of Urology, Shanghai Children's Medical Center Shanghai Jiao Tong University School of Medicine Shanghai China

**Keywords:** CRISPR‐Cas9, gene activation, human foreskin fibroblasts, induced Leydig cells, male hypogonadism, reprogramming

## Abstract

Recently, Leydig cell (LC) transplantation has been revealed as a promising strategy for treating male hypogonadism; however, the key problem restricting the application of LC transplantation is a severe lack of seed cells. It seems that targeted activation of endogenous genes may provide a potential alternative. Therefore, the aim of this study was to determine whether targeted activation of *Nr5a1*, *Gata4* and *Dmrt1* (NGD) via the CRISPR/dCas9 synergistic activation mediator system could convert human foreskin fibroblasts (HFFs) into functional Leydig‐like cells. We first constructed the stable Hsd3b‐dCas9‐MPH‐HFF cell line using the Hsd3b‐EGFP, dCas9‐VP64 and MS2‐P65‐HSF1 lentiviral vectors and then infected it with single guide RNAs. Next, we evaluated the reprogrammed cells for their reprogramming efficiency, testosterone production characteristics and expression levels of Leydig steroidogenic markers by quantitative real‐time polymerase chain reaction or Western blotting. Our results showed that the reprogramming efficiency was close to 10% and that the reprogrammed Leydig‐like cells secreted testosterone rapidly and, more importantly, responded effectively to stimulation with human chorionic gonadotropin and expressed Leydig steroidogenic markers. Our findings demonstrate that simultaneous targeted activation of the endogenous NGD genes directly reprograms HFFs into functional Leydig‐like cells, providing an innovative technology that may have promising potential for the treatment of male androgen deficiency diseases.

## 1 INTRODUCTION

Leydig cells (LCs), which are distributed in the interstitial tissue of the testis, are the primary source of androgens and play an essential role in spermatogenesis and male development.^1^, ^2^ Male hypogonadism may be associated with symptoms related to low production of testosterone, such as changes in body composition, increased fatigue, sexual dysfunction, depressed mood and decreased bone mineral density.^3^, ^4^, ^5^ Traditional testosterone replacement therapy has a good effect on male hypogonadism. However, the application of this treatment is limited because of side effects such as increased cardiovascular and prostate complications.^6^

As a better alternative, LC transplantation could result in the production of testosterone for a longer time period and maintain physiological patterns of the hormone.^7^ However, the real problem restricting the application of LC transplantation is that there is a severe lack of seed cells.^1^ In past decade, stem cells from different sources have been induced to differentiate into LCs.^8^, ^9^, ^10^, ^11^, ^12^ Our previous studies have demonstrated that umbilical cord‐derived mesenchymal stem cells (MSCs) can by differentiated into LCs by appropriate inducing factors.^13^, ^14^ However, MSCs may present a low differentiation efficiency, ethical concerns and even tumorigenic risk. Consequently, the identification of an alternative source of LCs would be of extraordinary significance for clinical applications as well as basic research.

In recent years, a new strategy for the direct and rapid manipulation of cell fates has emerged.^15^, ^16^ Researchers have made attempts to convert fibroblasts from various sources into a variety of functional cell types.^17^, ^18^, ^19^ Yan et al successfully reprogrammed mouse fibroblasts into induced Leydig cells (iLCs) by introducing multiple exogenous genes.^20^ This strategy will provide a useful reference for reprogramming human cells into iLCs by a different strategy.

Advances in genome editing techniques have made revolutionized both basic research and translational medicine. Recently, the discovery of the CRISPR/Cas9 system has promoted the development of related tools.^21^ The original attempts to develop CRISPR/Cas9 into a transactivator were realized by the a transactivation domain VP64 to dCas9.^22^, ^23^ The initial version of the technology had a limited capacity for target activation of endogenous genes using only a single guide RNA (sgRNA). To improve the efficiency, the dCas9/sgRNA complex, consisting of dCas9‐VP64 and synergistic activation mediator (SAM), was applied in this study.^24^, ^25^, ^26^ The second‐generation CRISPR/dCas9 systems proved to be efficient in functional genetic studies in vitro.^27^, ^28^

Several groups have taken advantage of CRISPR/dCas9 to direct the differentiation of stem cells.^24^, ^29^, ^30^ However, whether this new technology can reprogram human fibroblasts into LCs has not been studied previously. In recent years, some studies have confirmed that the combination of three key transcription factors (TFs), NR5A1, GATA4 and DMRT1, screened from a number of factors, can reprogram fibroblasts into iLCs.^20^, ^31^ Therefore, we hypothesized that targeted activation of these three key TFs simultaneously could convert human foreskin fibroblasts (HFFs) into functional iLCs.

To test this hypothesis, in this study, we first constructed a stable Hsd3b‐dCas9‐MPH‐HFF cell line including a fluorescent reporter system. Next, we generated a sgRNA lentivirus and used it to infect the constructed cell lines. Finally, we successfully obtained reprogrammed cells that produced testosterone as well as LC lineage markers. Thus, the iLCs obtained using the CRISPR/Cas9 SAM system may present a potential therapeutic value for treating male hypogonadism in the future.

## 2 materials and methods

### 2.1 Cell culture

Primary HFFs were isolated and cultured according to our previous studies.^31^ In brief, after informed consent was obtained from the patient's guardian, healthy boy's foreskin tissues from the phimosis operation were collected according to the guidelines of the World Medical Association Declaration of Helsinki and washed with phosphate buffer saline (PBS) (HyClone, Logan, UT, USA) including 2% penicillin‐streptomycin (HyClone) several times. The subcutaneous connective tissue was dissected away, and the remaining tissue was then minced into 2‐3 mm pieces with sterile ophthalmic scissors, after which the samples were digested with collagenase type I (Slorbia, Beijing, China) solutions (1‐2 mg/mL). The cells were dispersed through a sterile cell strainer (70 μm, Corning, Acton, MA, USA) after incubation at 37°C for 4 to 18 hours. The dispersed cells were washed several times via centrifugation (250 × g for 5 minutes) in PBS, and the cell pellet was resuspend in DMEM/high‐glucose medium (Gibco, USA) containing 10% foetal bovine serum (FBS, HyClone) and 1% penicillin‐streptomycin. Subsequently, the cells were plated in 10 cm cell culture dishes (Corning) and grown in medium for 2‐3 days at 37°C under 5% CO_2_. The culture medium in the dishes was replaced with fresh medium every 2 to 3 days. The first three generations of fibroblasts were selected for subsequent experiments.

### 2.2 Plasmid design and construction

**Figure 1:**
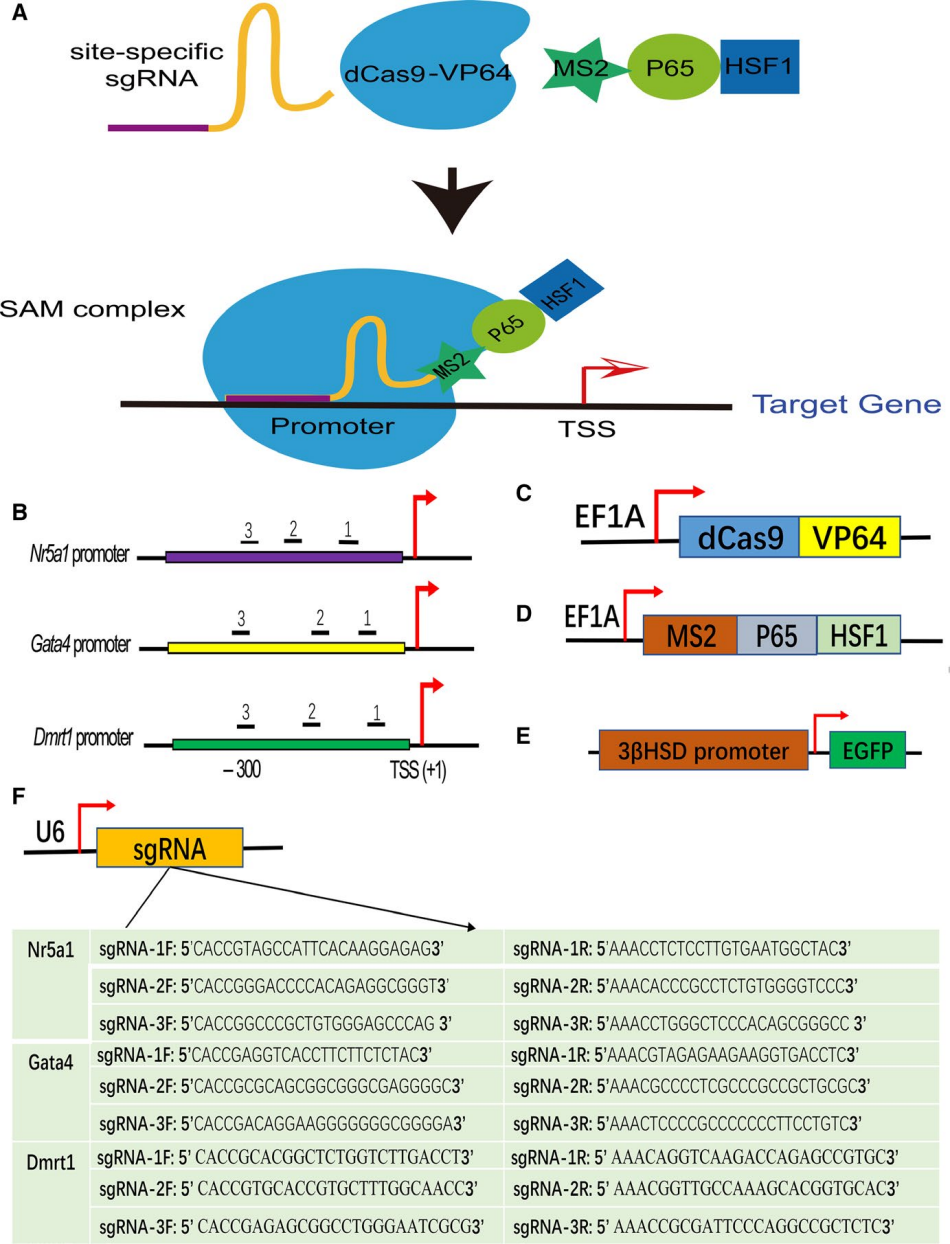
The single guide RNA (sgRNA) mediated CRISPR/dCas9 synergistic activation mediator (SAM) system for target gene activation. A, Schematic diagram of target gene activation, indicating that the SAM complex, consisting of three components, binds to the endogenous target gene promoter and transcriptional activation occurs. B, Three sgRNAs targeted each of the endogenous human *Nr5a1*, *Gata4* and *Dmrt1* gene promoters. TSS, transcription start site. C, Schematic representation of lenti‐dCas9‐VP64 plasmids expressing dCas9‐effector fusion proteins. D, Schematic representation of lenti‐MS2‐P65‐HSF1 (MPH) plasmids expressing the transcriptional activation complex. E, The lenti‐3βHSD‐EGFP plasmid reporter system in which 3βHSD gene promoter fragments were cloned upstream of EGFP. F, Schematic representation of the Lenti sgRNA(MS2)‐Puro vector. For the cloning of these designed sgRNAs, the insertion site of the guidance sequence lies downstream of the U6 promoter

To improve transcriptional activation, sgRNAs were designed to target the first 400 bp upstream of the target gene transcription start site (TSS) (Figure 1B). Optimal target sequences were determined on the basis of predicted off‐target scores using the online design tool at http://crispr.mit.edu, which was developed by the Zhang lab.^32^ The mock‐sgRNA (sgMock) target sequence was synthesized as described previously.^33^ Three guides on the sense strand were considered and subsequently cloned into the lenti‐sgRNA (MS2)‐Puro backbone (Genomeditech Co. Ltd., Shanghai, China) at the BbsI site. The final constructs were verified by clone sequencing. The primers are shown in Figure 1F.

Lenti‐dCas9‐VP64_Blast and lenti‐MS2‐P65‐HSF_Hygro were obtained from Addgene (#61425 and #61426), and the sequence of the Hsd3b promoter driving enhanced green fluorescent protein (EGFP) was cloned into the pCDH‐CMV‐MCS‐EF1‐Puro vector (TranSheepBio, Shanghai, China) using the Spel or EcoRI site; all of these constructs were confirmed by sequencing.

### 2.3 Lentiviral production

NIH 293T cells were cultured in DMEM/high‐glucose medium containing 10% FBS, and the cultures were split and seeded into 10 cm culture dishes. The next day, these lentiviral vectors and two other homologous helper plasmids were cotransfected into cells using the FuGENE® 6 transfection reagent (Promega, Wisconsin, USA) according to the manufacturer's instructions. Individual supernatants containing virus were harvested 48 hours or 72 hours after transfection and were concentrated with a centrifugal ultrafiltration device (Amicon Ultra 15 mL 100 K, Millipore, USA).

### 2.4 Establishment of stable cell lines and infection with sgRNAs

**Figure 2:**
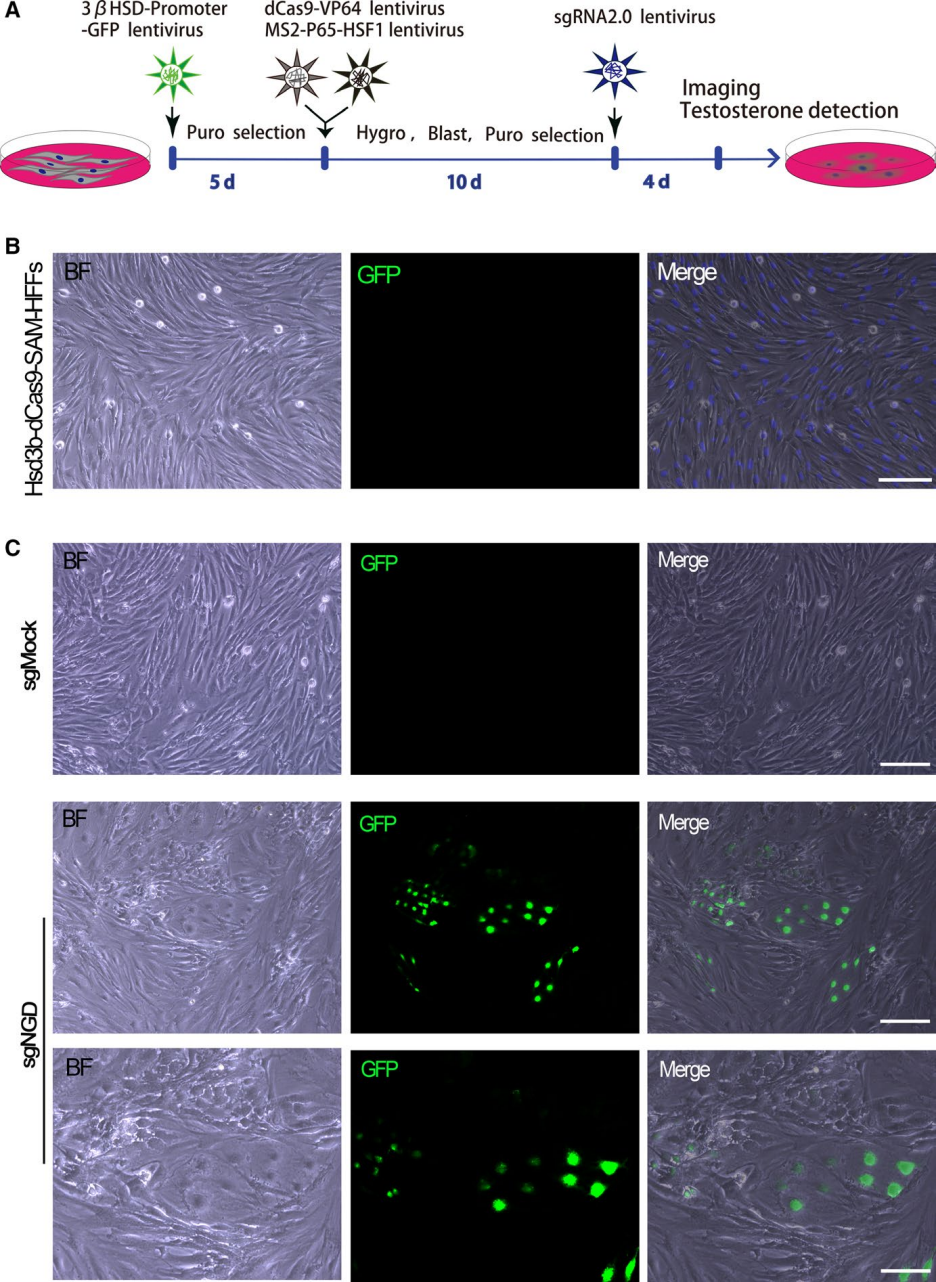
Generation of Hsd3b‐GFP‐positive cells after infection with sgRNA lentiviral vectors. A, Schematic diagram of the experimental procedure. B, Representative microscopy images of the stable Hsd3b‐dCas9‐MPH‐HFF cell lines constructed as described in Figure 1A. Human foreskin fibroblasts were infected with the indicated lentivirus at an MOI of 20, and at day 10 after infection with dCas9‐vp64 and MPH lentivirus, samples were stained with Hoechst 33342 (blue). Scale bars = 100 μm. C, Representative microscopy images showing the cellular appearance at day 4 after sgRNA transduction (MOI = 20). Scale bars = 100 μm (upper panel) or 50 μm (lower panel)

To establish Hsd3b‐dCas9‐MPH‐HFF cell lines with stable expression, HFFs were plated in 100 mm dishes (0.5‐1.0 × 10^6^ cells/dish) and then infected with the indicated lentiviral supernatants with the polybrene (final concentration: 10 μg/mL) (Santa Cruz) on the next day. The culture supernatants were replaced with fresh medium containing antibiotics 48 hours after transduction. The total period of antibiotic selection was not less than 2 weeks, as shown in Figure 2A. The optimized antibiotic concentrations were as follows: puromycin (1.5 μg/mL), blasticidin S (30 μg/mL) and hygromycin B (50 μg/mL) (all antibiotics from Yeasen, Shanghai, China). Furthermore, the established cell lines were stained with Hoechst 33342 (15 μg/mL, Yeasen) and observed under a fluorescence microscope (Leica DMi8, Germany).

After successful establishment of the stable cell lines, the cells were treated with the indicated lentiviral vectors encoding the corresponding sgRNA at a multiplicity of infection (MOI) of 20. To activate the three endogenous target genes simultaneously, the ratio of sgRNAs was 1:1:1. Forty‐eight hours postinfection, the culture supernatants were replaced with fresh medium, and the cells were observed using a fluorescence microscope at day 4 after infection.

### 2.5 Flow cytometry analysis and cell sorting

Four days after infection with sgRNAs, cells were harvested using 0.25% trypsin (Thermo Fisher Scientific), washed once with 1 × PBS and resuspended with PBS. At least 10 000 living cells were analysed on a flow cytometer equipped with BD FACSDiva software. The Hsd3b‐dCas9‐MPH‐HFFs were used as isotype controls. For purification of the Hsd3b‐GFP^+^ cells, the cells were treated as described above and sorted with a FACS instrument (MoFlo XDP, Beckman Coulter, Brea, CA, USA).

### 2.6 RNA extraction and quantitative real‐time polymerase chain reaction (qRT‐PCR)

Total RNA was extracted from small cell samples using the UNlQ‐10 Total RNA Isolation Kit (Sangon Biotech, Shanghai, China) according to the manufacturer's guidelines, and the concentration of RNA was determined with a NanoDrop2000 spectrophotometer (Thermo Fisher Scientific, USA). Total RNA was treated with gDNA Remover (Sangon) and used for cDNA synthesis (Bio‐Rad) using the PrimeScript™ RT reagent Kit (RR037A, Takara, China). Real‐time PCR (CFX384 system, Bio‐Rad) was performed using POWRUP SYBR MASTER MIX (Thermo Fisher Scientific) in a total volume of 10 μL. The relative expression levels of mRNA were normalized to the corresponding GAPDH values and shown as the fold change relative to the sgMock control. The relative gene expression level was calculated using the 2^‐ΔΔCt^ method. The corresponding primers are listed in Table S1.

### 2.7 Total protein extraction and Western blotting

To extract whole‐cell proteins, cells were lysed in 1 × RIPA buffer (Solarbio, Beijing, China) containing a cocktail solution of protease inhibitors (Sangon Biotech) on ice for 30 minutes and then centrifuged (12 000 × g for 10 minutes at 4°C). Subsequently, the Bradford method was used to normalize protein concentrations, and a representative Western blotting protocol was adopted. In brief, 20 μg of protein per channel was separated through 10% SDS‐PAGE and then transferred to polyvinylidene difluoride (PVDF) membranes (Millipore, Billerica, MA, USA). After blocking with a blocking solution (5% skim milk in Tris‐buffered saline with 0.1% Tween‐20, TBS‐T) for 1 hour, the membranes were incubated with the primary antibodies (including rabbit anti‐human NR5A1: ab168380, GATA4: ab134057, DMRT1: ab126741, GAPDH: ab37168, Abcam, Cambridge, UK, at 1:2000; and rabbit anti‐human CYP17A1: orb213833, HSD3B1: orb5478, STAR:orb129747, CYP11A1: orb213832, Biorbyt, San Francisco, CA, USA, at 1:1000) overnight at 4°C. Next, the membranes were incubated with the goat anti‐rabbit secondary antibody (ab150079, Abcam, 1:10000) for 1 hour at RT in the dark, and specific bands were detected using a two‐colour Infrared Laser Imaging System (LI‐COR Odyssey, USA).

### 2.8 Immunofluorescence staining

For immunofluorescence analysis, cells were fixed with 4% paraformaldehyde (PFA) solution at 4°C for 30 minutes and then exposed to 0.3% Triton X‐100 (Sigma, USA) PBS solution for 5 minutes at room temperature (RT). Subsequently, the cells were blocked with 5% BSA in PBS for 30 minutes and then incubated with the primary antibody (CYP17A1: orb213833, STAR: orb129747, CYP11A1: orb213832, Biorbyt; 1:200) for 1 hour at RT. After rinsing several times, the cells were incubated with a goat anti‐rabbit secondary antibody (ab150079, Abcam; 1:500) for 1 hour at RT in the dark. Finally, nuclei were stained with Hoechst 33342 (15 μg/m, Yeasen) and then examined under a fluorescence microscope (Leica DMi8, Germany).

### 2.9 Testosterone concentration assay

The culture medium supernatant was collected at the specified time points, and the concentrations of testosterone were assayed at Shanghai Children's Medical Center by chemiluminescence using the Access Testosterone Kit (REF 33560, Beckman Coulter, Brea, CA, USA) according to the manufacturer's guidelines. In brief, the contents of new reagent packs were mixed by gently inverting the packs several times before loading into the instrument. A 20 µL aliquot of each sample was used for each determination in addition to the sample container and system dead volumes, and measurements were performed automatically by the UniCel DxI 800 system (Beckman Coulter). The detection limits of this system are approximately 0.1‐16 ng/mL. The inter‐assay and intra‐assay variations were less than 10% and 5%, respectively.

### 2.10 Chromatin immunoprecipitation quantitative PCR (ChIP‐qPCR)

A modified ChIP protocol based on previous reports^34^, ^35^ was performed. In brief, cells were treated with PBS containing 0.75% formaldehyde at RT for 10 minutes. Glycine was added to a final concentration of 125 mM, followed by incubation for 10 minutes. After rinsing the samples twice with 5 mL of cold PBS, the samples were harvested by scraping, centrifuged (1000 × g, 4°C, 5 minutes) and resuspended in 700 μL of cold ChIP lysis buffer. Subsequently, the samples were sonicated with an ultrasonic crusher (Sonics VCX750, USA) 10 times for 10 seconds each time at intervals of 50 seconds. The samples were centrifuged (8000 × g, 4°C, 10 minutes) again, and the supernatants were diluted 1:10 in ChIP dilution buffer. Non‐specific background was removed by adding a protein A‐agarose/salmon sperm slurry. One‐tenth of the volume of the supernatants was stored as an input sample, while the remainder was incubated continuously for 12 hours with 2 μg of the corresponding antibodies at 4°C with rotation. The immunocomplexes were harvested with 60 μL of salmon sperm DNA/protein A/G‐agarose (26156, Thermo Fisher Scientific, USA) at 4°C for 3 hours with rotation. The beads were washed sequentially with the following buffers: IP wash buffer 1 (once), IP wash buffer 2 (twice) and IP wash buffer 3 (once). Subsequently, the immunocomplexes were eluted by the addition of 150 μL of IP elution buffer to the beads at RT for 20 minutes with vortexing and incubated overnight at 65°C with shaking. The DNA was recovered through reverse cross‐linking with isoamyl alcohol‐chloroform‐phenol (1:24:25) extraction and ethanol precipitation. ChIP‐grade H3K27ac (ab4729, Abcam) and 3K4me3 (ab213224, Abcam) antibodies were used. qPCR was performed using POWRUP SYBR MASTER MIX (Thermo Fisher Scientific), and the data are shown as the fold change relative to the negative control (IgG‐bound DNA), normalized to GAPDH. The ChIP‐qPCR primers are listed in Table S2.

### 2.11 Statistical analysis

All of the data are shown as the mean ± SD. The significance of a difference between groups was assessed via the two‐tailed Student's *t* test using Prism 6 software (GraphPad Software, Inc, La Jolla, CA, USA). A value of *P* < 0.05 was considered statistically significant.

## 3 RESULTS

### 3.1 Design of the NGD‐targeting CRISPR/dCas9 SAM system

The Nr5a1, Gata4 and Dmrt1 genes, encoding TFs have been confirmed to be crucial in LC specification and differentiation. Therefore, in this study, we decided to activate these endogenous target genes simultaneously in HFFs using the powerful CRISPR/dCas9 SAM system (Figure 1A). It has been reported that targeting the promoter region, which is close to the transcription start site, can lead to maximum gene activation.^25^ We therefore designed three lentiviral plasmids containing the U6 promoter for sgRNAs per gene; the encoded sequences bound to three separate target regions within the −369 to +1 upstream region of the TSS of the target gene (Figure 1B, 1). In addition to the dCas9‐VP64 lentiviral vector expressing dCas9‐effector fusion proteins (Figure 1C), we took advantage of MS2‐P65‐HSF1 (MPH) lentiviral vectors expressing the transcriptional activation complex (Figure 1D). Hsd3b encodes the 3β‐hydroxysteroid dehydrogenase (3β‐HSD) that is electively expressed in gonadal steroidogenic cells. To monitor efficient cell fate conversion in reprogramming studies, we also successfully constructed an Hsd3b‐EGFP lentiviral vector (Figure 1E), in which Hsd3b gene promoter fragments were cloned upstream of EGFP.

### 3.2 Conversion of HFFs into Hsd3b‐GFP‐positive cells via dCas9‐mediated multiple gene activation

To quickly analyse the reprogramming efficiency and separate Hsd3b‐GFP‐positive cells from HFFs by fluorescence‐activated cell sorting (FACS), we established an Hsd3b‐EGFP reporter system by constructing Hsd3b‐dCas9‐MPH‐HFF cell lines. To accomplish this, we transduced a lentiviral vector carrying the EGFP gene driven by the Hsd3b promoter into HFFs expressing dCas9‐effector fusion proteins and the transcriptional activation complex simultaneously (Hsd3b‐dCas9‐MPH‐HFFs), followed by selection with puromycin, blasticidin S, and hygromycin B over 15 days (Figure 2A). After lentivirus transfection and antibiotic selection, no Hsd3b‐GFP^+^ cells were detected by fluorescence microscopy (Figure 2B), showing that the stable Hsd3b‐dCas9‐MPH‐HFF cell line was established successfully.

In subsequent studies, the Hsd3b‐dCas9‐MPH‐HFFs were lentivirally transduced with sgMock and the combination of sgNr5a1, sgGata4 and sgDmrt1 (sgNGD) (MOI = 20). At day 4 post‐transduction with sgRNAs, some of these cells infected with sgNGD exhibited remarkable morphological changes compared with the sgMock control, and the morphologically altered cells intriguingly expressed the GFP marker; however, this phenomenon was not detected in other cells (Figure 2C). These results suggested that HFFs were converted into Hsd3b‐GFP‐positive cells using the CRISPR/dCas9 SAM system.

### 3.3 Characteristics of Hsd3b‐GFP^+^ cells converted from HFFs

**Figure 3:**
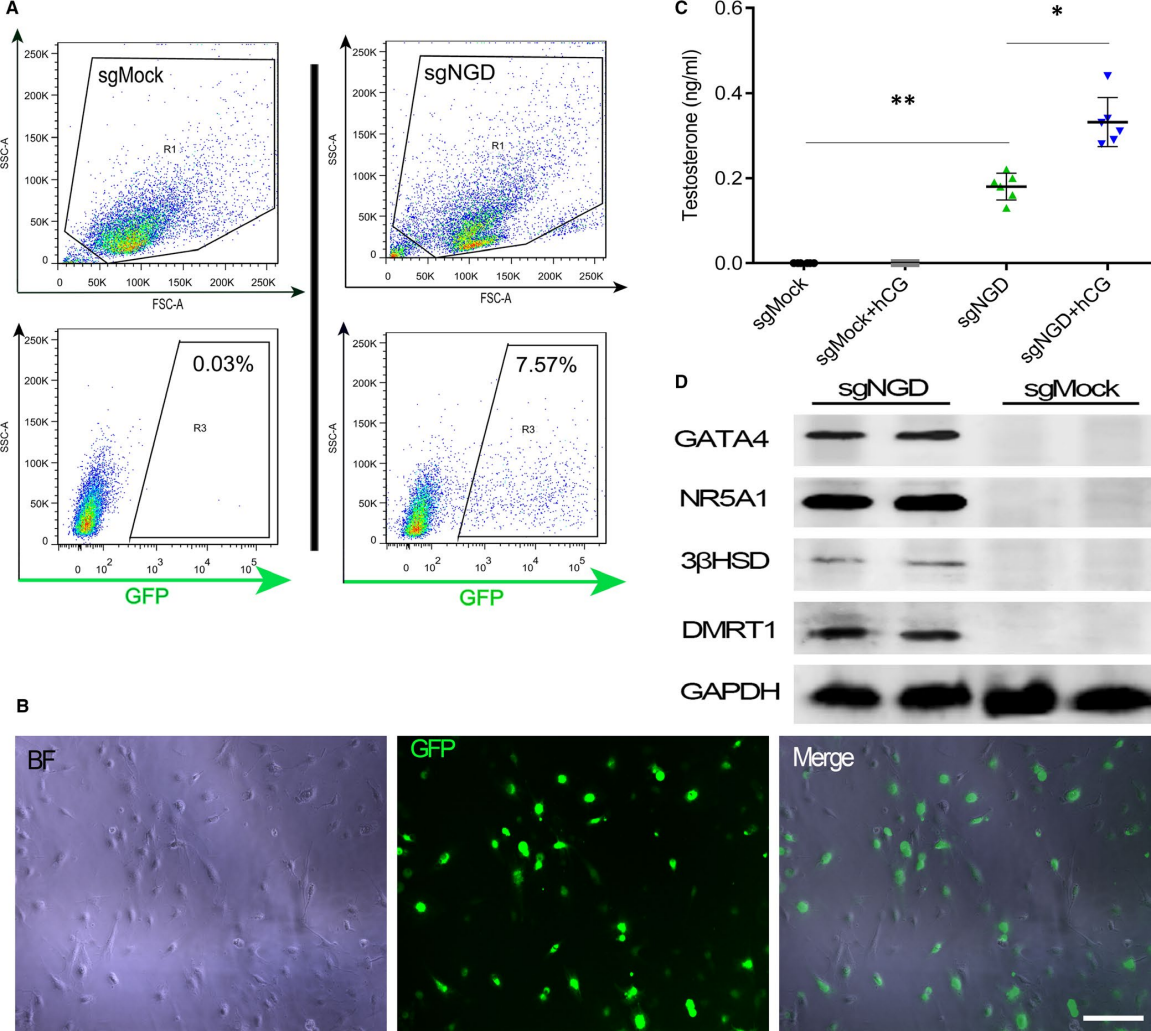
Preliminary characteristics of Hsd3b‐GFP^+^ cells converted from human foreskin fibroblasts (HFFs). A, Representative photographs of Hsd3b‐GFP^+^ cells purified by fluorescent‐activated cell sorting (FACS) at 4 d after infection with the sgNGD lentivirus. B, Representative FACS plots of Hsd3b‐dCas9‐MPH‐HFFs at day 4 after treatment with the sgRNA lentivirus. Scale bars: 100 μm. C, Testosterone production in cells transduced with sgNGD and sgMock. At day 4, the cell medium was replaced by fresh medium containing hCG (10 ng/mL) for 24 h, after which hormones in the culture supernatant were assayed. **P < 0.01, *P < 0.05, significant difference compared to the sgMock control. Data are presented as the mean ± SD (n = 6 independent experiments). D, Representative western blotting results for the expression of transcription factors related to steroidogenic markers in 3βHSD‐GFP^+^ cells at day 14 after infection

To test the reprogramming efficiency of HFFs, after treatment with sgMock and sgNGD, the cells were continually cultured and then sorted by FACS based on green fluorescence at day 4 postinfection (Figure 3A). The results suggested that there were approximately 757 reprogrammed cells among every 10 thousand cells treated with sgNGD. Subsequently, these cells isolated from HFFs were plated on gelatin‐coated dishes and further cultured. One day after culture, almost all cells adhered to the bottom of the dish, exhibited an ovoid‐like morphology similar to adult LCs and persistently expressed the Hsd3b‐GFP marker (Figure 3B). We next added the hCG hormone to the culture medium at a final concentration of 10 ng/mL in the sgMock and sgNGD treatments for an additional 24 hours and then examined testosterone production. Cells treated with sgNGD or sgNGD+hCG produced testosterone at mean levels of 0.20 and 0.33 ng/mL, respectively, while testosterone was not detected in the sgMock control or sgMock+hCG group; moreover, a significant increase in testosterone was observed in sgNGD+hCG compared with sgNGD (Figure 3C). These reprogrammed cells were able to secrete testosterone and respond effectively to stimulation by the hCG hormone.

To understand the expression levels of target endogenous genes, we performed Western blotting on cells treated with sgRNAs, and protein expression of the NR5A1, GATA4 and DMRT1 TFs and downstream HSD3b was detected in the sgNGD group, but not in the sgMock control (Figure 3D). Taken together, the above data demonstrated that a set of three sgRNAs targeting each of the three TFs NR5A1, GATA4 and DMRT1 achieved simultaneous activation of the endogenous genes in HFFs and that Hsd3b‐GFP^+^ cells were reprogrammed successfully and acquired initially the endocrine function of LCs.

### 3.4 Hsd3b‐GFP^+^ cells exhibit partial characteristics of LCs

**Figure 4:**
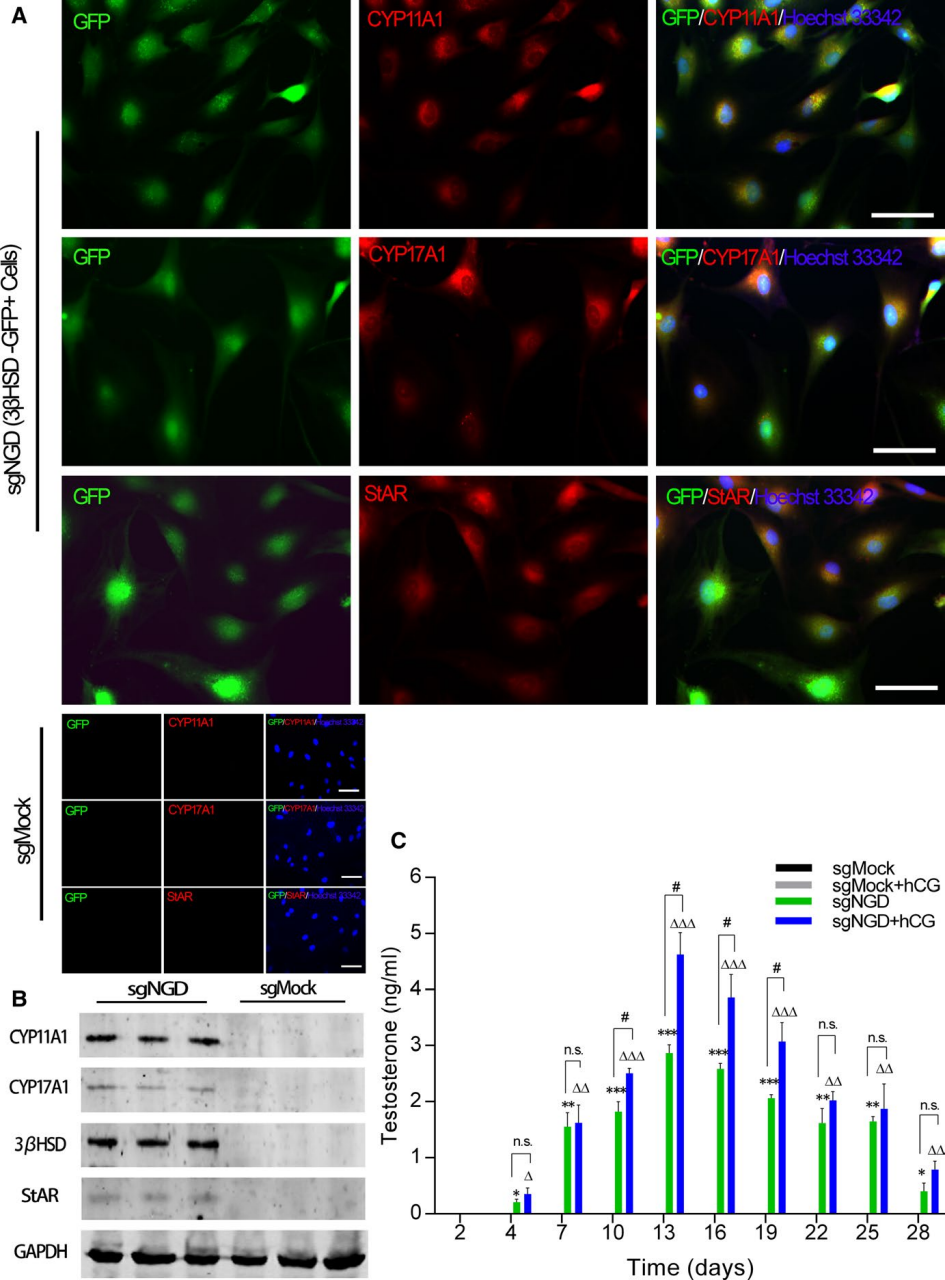
Further characterization of Hsd3b‐GFP^+^ cells. A, Immunofluorescence staining revealed the expression of steroidogenic enzymes at day 14 postinfection of the sgNGD lentivirus. Hoechst 33342 was used to counterstain nuclei (blue). Scale bars = 50 µm. B, Representative Western blotting results for the protein expression of Leydig steroidogenic markers in induced Leydig‐like cells at day 14 postinfection with the sgNGD lentivirus. C, Time course of testosterone production during culture after infection with the sgRNA lentivirus. Every 3 days, the testosterone concentration in the supernatant was measured, and the supernatant was replaced by fresh medium either containing hCG (10 ng/mL) or not. *P < 0.05, **P < 0.01, ***P < 0.001, significant difference compared to sgMock; ^△^ *P* < 0.05, ^△△^ *P* < 0.01, ^△△△^ *P* < 0.001, significant difference compared to sgMock + hCG; ^#^ *P* < 0.05, significant difference between the indicated groups; ns, not significant. Data are presented as the mean ± SD of 3 biological replicates

To assess the expression levels of downstream Leydig steroidogenic markers including steroidogenic acute regulatory protein (StAR), cytochrome P45017A1 (CYP17A1) and cytochrome P450 cholesterol side chain cleavage (CYP11A1) in the induced Leydig‐like cells (iLCs), these cells were isolated by FACS at day 4 postinfection and then seeded into 6‐well plates for further culture; 10 days later, we performed Western blotting as well as immunofluorescence staining on the Hsd3b‐GFP^+^ and sgMock control cells. Subsequently, immunofluorescence staining showed that the sgNGD‐induced Leydig‐like cells expressed the LC lineage markers CYP11A1, CYP17A1 and StAR, while the sgMock‐induced HFFs did not express these steroidogenic enzymes (Figure 4A). Western blotting results also validated the above markers in sgNGD‐induced Leydig‐like cells (Figure 4B).

Furthermore, to analyse the characteristics of testosterone production in the iLCs, sorted Hsd3b‐GFP^+^ cells were plated in DMEM/high‐glucose medium supplemented with 10 ng/mL hCG at a density of 1 × 10^6^ cells per well. At the indicated time points, the supernatants were collected for quantitative determination of testosterone and were replaced with fresh medium. The data obtained from quantitative determination revealed that the iLCs began to produce testosterone at day 4 posttransduction; moreover, the yield progressively increased, peaking at 2.89 ± 0.21 ng/mL or 4.62 ± 0.61 ng/mL at day 14 after transduction, and declined thereafter (Figure 4C). Interestingly, the concentration of testosterone produced by the hCG‐stimulated iLCs was higher than that produced by basal iLCs, and the difference between these groups was significant (*P* < 0.05) (Figure 4C).

**Figure 5:**
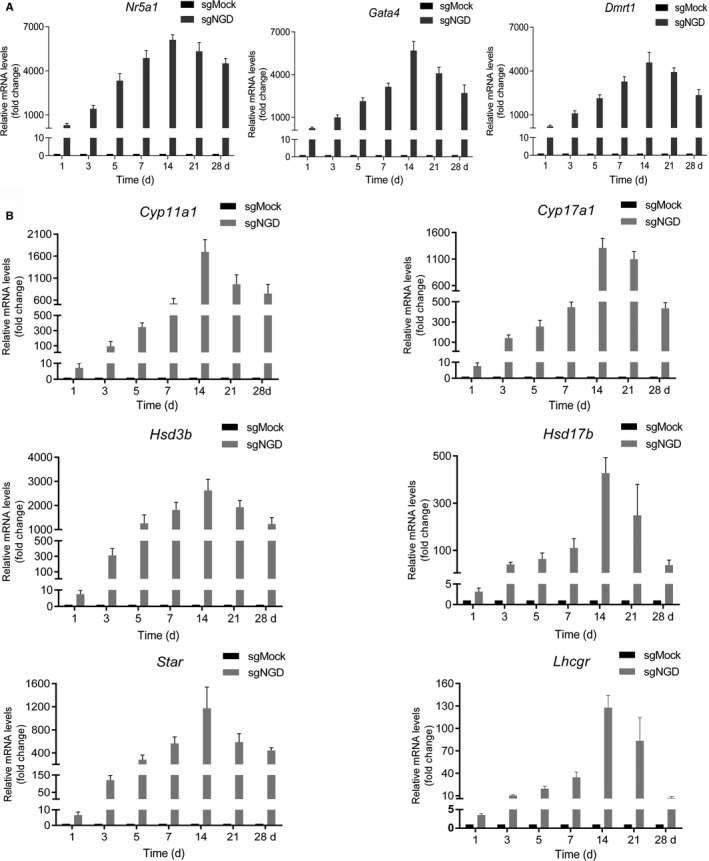
Time course of corresponding gene expression. A, B, RNA was obtained from the cells infected with the sgMock or sgNGD lentivirus on the indicated day. Relative mRNA levels of the indicated genes were measured by quantitative real‐time polymerase chain reaction. The relative mRNA levels were calculated using the delta‐delta Ct method：fold change = 2^‐ΔΔCt^. Relative mRNA levels were normalized against those of GAPDH and then standardized to t in the sgMock control sample. Data are presented as the mean ± SD of 3 biological replicates

We next examined the expression levels of genes including *Nr5a1*, *Gata4* and *Dmrt1* by qRT‐PCR analysis at the indicated time points, and the relative quantitative data revealed that the mRNA levels in cells treated with sgNGD were higher than those in cells treated with the sgMock control (Figure 5A). We further evaluated the kinetics of the expression of downstream Leydig steroidogenic markers including *Cyp11a1*, *Cyp17a1*, *Hsd3b*, *Hsd17b*, *Star* and *Lhcgr* and the up‐regulation of these specific markers was subsequently validated by qRT‐PCR in sgNGD‐treated cells (Figure 5B). In addition, the trend of the time course of corresponding gene expression was consistent with the characteristics of testosterone production (Figure 4C and 5A, 5), which showed a positive correlation between testosterone production and the level of target gene activation. These results indicated that iLCs rapidly acquired the ability to secrete testosterone following transduction, though it took some time to reach the peak concentration, and more importantly, the cells responded to hCG in vitro.

### 3.5 In vitro activation of target genes in HFFs by the CRISPR/dCas9 system promotes epigenetic remodelling

**Figure 6:**
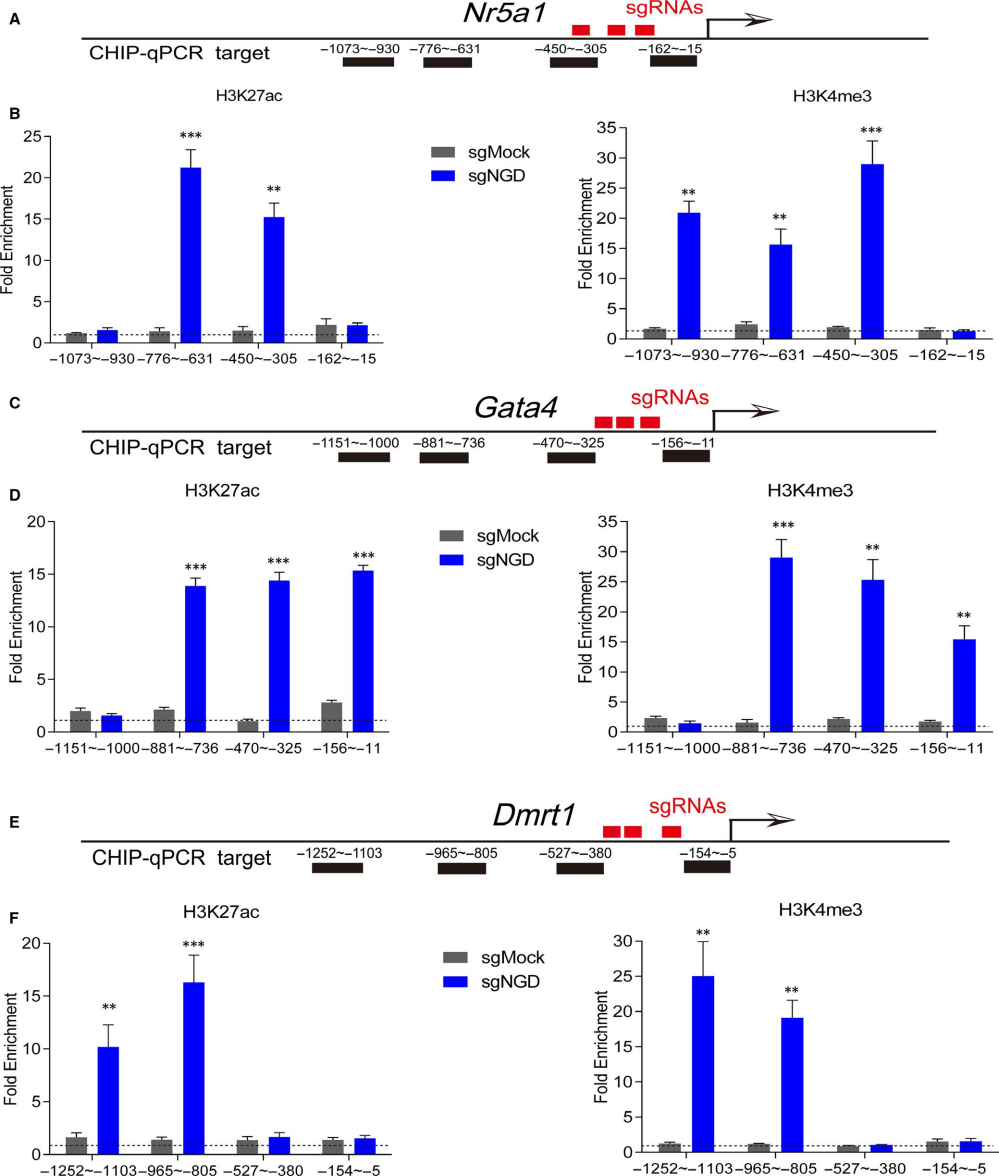
In vitro, the CRISPR/dCas9 system leads to rapid epigenetic remodelling at target loci. A, C, E, Schematic representation of the target genomic loci. The sgRNA target sites upstream of the transcription start site (red bars) and the ChIP‐qPCR regions are indicated (black bars). B, D, F, Activation of the endogenous targets Nr5a1, Gata4 and Dmrt1 in human foreskin fibroblasts resulted in significant enrichment of H3K4me3 and H3K27ac at multiple loci at day 4 after infection (**P < 0.01, ***P < 0.001 n = 3 biological replicates). All fold enrichments are relative to IgG (negative control) and normalized to GAPDH

Next, we sought to determine whether the dCas9‐mediated activation of target genes could result in rapid epigenetic remodelling at the corresponding loci. To answer this question, we performed ChIP‐qPCR in Hsd3b‐dCas9‐MPH‐HFFs treated with the sgMock or sgNGD lentiviral vectors at day 4 posttransduction. We used qPCR primers (Figure 6A, 6C, 6E) tiled along the promoter regions of the *Nr5a1*, *Gata4* and *Dmrt1* loci and subsequently detected obvious enrichment of H3K4me3 and H3K27ac at the indicated gene loci in the sgNGD‐treated cells (Figure 6B, 6D, 6F). In contrast, in the sgMock‐treated cells, there was no clear detectable change in H3K4me3 or H3K27ac within the corresponding putative *Nr5a1*, *Gata4* and *Dmrt1* promoters. These results revealed that the CRISPR/dCas9 SAM system activated an endogenous gene that was normally silent via trans‐epigenetic remodelling.

## 4 DISCUSSION

In this study, we demonstrated that direct cellular conversion into iLCs was achieved via simultaneous targeted activation of three endogenous genes. First, we successfully constructed a stable Hsd3b‐dCas9‐MPH‐HFF cell line harbouring a GFP reporter system. Furthermore, we used the CRISPR/dCas9 SAM system based on a locus‐specific regulator of transcriptional activation for multiplex target activation of the steroidogenic factors *Nr5a1*, *Gata4* and *Dmrt1* (NGD factors). To improve the activation of target genes, we designed 3 sgRNAs for every target gene.^36^, ^37^ In contrast to the sgMock control, the cells infected with sgNGD expressed GFP and produced testosterone at day 4 posttransduction, in addition to exhibiting clear morphologic changes. These results showed that targeted activation of endogenous genes requires an extra day to achieve reprogramming compared with ectopic overexpression of the exogenous reprogramming factors.^20^ The main cause of the above difference could be that the former approach involves one more step than the latter during reprogramming,^16^ or it might be due to the different species involved.

To further evaluate the reprogrammed Hsd3b‐GFP^+^ cells, we purified these cells using FACS at day 4 after infection with sgRNAs. The flow cytometric analysis showed that the reprogramming efficiency was approximately 7.57%, which was higher than that for induced human MSCs, though it was not higher than that associated with ectopic overexpression of the exogenous reprogramming factors.^31^ The main reason for this difference may be the different species involved. In general, for cells from the same species, the reprogramming efficiency based on CRISPR/dCas9 is higher than that for traditional methods.^37^, ^38^ Several studies have indicated that the speed, efficiency and robustness of reprogramming may be restricted by the slow epigenetic remodelling process and divergence to a competing cell identity.^16^, ^39^, ^40^ Therefore, achieving robust desired populations of reprogrammed cells remains a major challenge. Interestingly, our studies demonstrated that these purified Hsd3b‐GFP^+^ cells retained a similar appearance to adult LCs and could secrete more testosterone upon stimulation with hCG. However, the Hsd3b‐GFP^+^ cells showed a relatively weak response to hCG, and it seemed that these cells still needed to develop further. This finding was in accord with a previous study.^20^ The results indicated that the reprogrammed cells preliminarily gained the endocrine function of LCs.

Subsequently, to further confirm the function of the Hsd3b‐GFP^+^ cells, we assessed the expression levels of steroidogenic markers at day 14 postinfection through Western blotting and immunofluorescence staining. As expected, these cells treated with sgNGD expressed steroidogenic enzymes such as CYP11A1, CYP17A1, HSD3B and StAR, which are selectively expressed in steroidogenic cells.^41^ These results suggested that multiplex activation of targeted *Nr5a1*, *Gata4* and *Dmrt1* could directly convert HHFs into functional iLCs.

In vitro, we found that the reprogrammed cells began to produce testosterone at day 4 post‐transduction, and the yield progressively increased, peaking as 2.89 ± 0.21 ng/mL for treatment with sgNGD alone and 4.62 ± 0.61 ng/mL for sgNGD with hCG (10 ng/mL) at day 14 after transduction, and declined thereafter. These results were similar to previous reports.^20^, ^31^ Therefore, the analyses revealed that the reprogrammed cells rapidly obtained the capacity to secrete testosterone, although time is required for optimal production to be achieved, and more importantly, the cells could respond to stimulation by the hCG hormone. Therefore, we speculate that the iLCs may secrete testosterone in physiological patterns under the control of the hypothalamus‐pituitary‐gonadal axis when they are transplanted into the body.

In addition, we found that in vitro, the trend of testosterone production was in accord with the trend of the gene expression of upstream target genes and downstream steroidogenic markers determined by qRT‐PCR. This finding may imply that the robustness of targeted activation via the CRISPR/dCas9 SAM system is maintained for only a limited period of time, similar to a previous report.^37^ There may be many factors involved in this situation, such as senescence of reprogrammed cells in the process of in vitro culture, non‐optimal culture conditions such as the absence of specific culture medium for LCs, off‐target effects in spite of a low probability and so on.^42^, ^43^, ^44^ However, the practical causes are largely unknown, and further studies are needed.

Several groups have found that the relevant mechanism of targeted activation of endogenous genes involves epigenetic remodelling.^35^, ^38^ To assess the effects of targeted activation on the epigenetic remodelling of chromatin at the target loci, we performed ChIP‐qPCR in Hsd3b‐dCas9‐MPH‐HFFs treated with sgNGD or sgMock. Our results showed that remarkable enrichment of H3K4me3 and H3K27ac at the *Nr5a1*, *Gata4* and *Dmrt1* loci was maintained at day 4 postinfection of sgNGD; however, the sgMock control did not show a significant change. H3K27ac and H3K4me3, both of which are epigenetic chemical modifications, are significantly associated with the transcriptional activation of nearby genes.^45^, ^46^ Therefore, we speculate that targeted transcriptional activation mediated by the CRISPR/dCas9 SAM system rapidly remodels their epigenetic signatures and facilitates the conversion of the cell lineage as an alternative strategy.

In summary, we have demonstrated a novel strategy for reprogramming HFFs to functional Leydig‐like cells through targeted activation of the endogenous genes *Nr5a1*, *Gata4* and *Dmrt1* using the CRISPR/dCas9 SAM system. The iLCs expressed steroidogenic enzymes and rapidly began to secrete testosterone. Encouragingly, the iLCs responded to stimulation by the hCG hormone, even though it did not seem that they responded optimally to hCG, and further optimization will be needed. Our strategy could alleviate the undesired effects of vector integration into the genome when adeno‐associated viruses (AAVs) or integrase‐deficient lentiviral vectors are used in the future, particularly considering that AAVs are preferred vectors for gene transfer in vivo due to their distinct advantages. Therefore, an innovative strategy based on endogenous target gene activation may provide a potential therapeutic alternative for male hypogonadism in the future.

## CONFLICT OF INTEREST

The authors declare no conflict of interest.

## AUTHORS CONTRIBUTIONS

HH and JS conceived and designed this study; HH, XYZ, LZ, YPH and JZ performed the research; HH and XYZ analysed the data; ZYZ and XYX contributed materials; HH and JS wrote the paper. All authors read and approved the content.
