# Supplementary material for: CRISPR/dCas9‐mediated activation of multiple endogenous target genes directly converts human foreskin fibroblasts into Leydig‐like cells
Source: J Cell Mol Med. 2019 Jul 2;23(9):6072–84. doi: 10.1111/jcmm.14470 (PMC6714237; doi:10.1111/jcmm.14470)
Supplement: Supplementary file 1 [file JCMM-23-6072-s001.docx]

SUPPLEMENTAL TABLES:

**Table S1.** **Primers for quantitative reverse transcription-polymerase chain reaction**

| **Gene** | **Sense primer** | **Antisense primer** |
| --- | --- | --- |
| *Nr5a1* | TCTGCAAGGTTGTAGTCAAGAG | CCCAGCTTCAGTCCTCAAAT |
| *Gata4* | AATCTTCCCTCTTCCCTCCTCAA | CAACACCCGCTTCCCCTAAC |
| *Dmrt1* | AGCTCATTGCATGGCGGTTCT | GCTGCTGATTCTGCTTCTGGG |
| *Lhcgr* | AGAGCCATCCTCCAAGCATAA | TCTACACCCTCACCGTCATCA |
| *Star* | GGCCTGAGAAGTCTTGCTTTAT | GACCCTCTGAGATTCTGCTTTG |
| *Cyp11a1* | AGGAAGTGTTCACCACGATTAC | CTGTGACGTTGGCCTTGAT |
| *Hsd3b* | TTTAGATTCCACCCATTAGCC | GCCAGTCTTCATCTACACCAG |
| *Cyp17a1* | GTCAAGGTGAAGATCAGGGTAG | CAAGATAGACAGCAGTGGAGTAG |
| *Hsd17b* | GATGATGACTTCTTTTGCTTTA | CTGAACATTTCTTCTGGGATA |
| *Gapdh* | CGCTGAGTACGTCGTGGAGTC | GCTGATGATCTTGAGGCTGTTGTC |

**Table S2. Primers for ChIP-qPCR within the promoter regions of target genes**

| **Target** | **Position** | **Sense primer** | | **Antisense primer** |
| --- | --- | --- | --- | --- |
| ***Nr5a1*** | -15 ～-162 | | AACTCAGGTGGGGGACAGAG | GCTGTGTGGGTCTGAGCCTA |
|  | -305 ～-450 | | AGGAGGCTGGCCATTAGAGG | CTCATCCGGTGTGAGAGCG |
|  | -631 ～-776 | | CTGTCCGTCCGTCCTCCTC | CGAGAGGCCTGCAGAGTCA |
|  | -930 ～-1073 | | GCCTGCTGCTTCCACCTCTT | GAACATCAGCAGCCCACAGC |
| ***Gata4*** | -11 ～ -156 | | GAGCTGGGGACTTGGAGGC | GTTCCGCGCACGCTCC |
|  | -325 ～ -470 | | GCCACCAGGTTGAGGAAAGG | GCTGCCCCAGGGATTCCA |
|  | -736 ～ -881 | | GCCAGCGAGGAGTGTCCTAT | TCAACCAATAGCCCTCTTCCGA |
|  | -1000～-1151 | | AAGGGAAAAGTTGGTGACCTCAG | TGGCAAATAGTTCATTCCAGCAAG |
| ***Dmrt1*** | - 5 ～-154 | | TGGATAACAGCAATTGACATGCG | ACAGGAGCTAGGATGCAACAA |
|  | -380 ～ -527 | | GCTCCAAAGGCAGATTCGAGG | CCAAAGAGGCATGGTCACCC |
|  | -805 ～ -965 | | GCTCAGCAGGCCTTAGGGATA | GCCTGTCCCAGGGTTCTCAT |
|  | -1103～-1252 | | AACACGGGCTGCAACAGTAG | TCCCTTCCTGCACCTGTCTT |
